# Advancing equity, one publication at a time

**DOI:** 10.1038/s41390-022-02102-y

**Published:** 2022-05-24

**Authors:** Elena Fuentes-Afflick

**Affiliations:** University of California, San Francisco, San Francisco, CA USA

Gender equity has become an important priority for society, including medicine and other health professions. Despite several decades of progress toward gender parity in medical school matriculation, training, and practice, there is a persistent lack of gender equity at senior rank and leadership positions.

Dissemination of new ideas, novel curricula, policy innovations, and scientific advances through publication is a well-established form of recognition and advancement. For physicians and researchers, publication is an essential requirement for advancement and is widely considered an indicator of success. In this issue, Bohme et al. published a comprehensive analysis of 10 years of English-language publications in pediatrics, which included many pediatric disciplines and multiple countries. The central question was whether there is gender equity in research engagement, as measured by publication. Based on a large sample of articles and authors, the authors reported that women were overrepresented as first authors and co-authors, relative to men, and underrepresented as senior authors. They computed the Prestige index, which compares the gender ratio at the more prestigious first and last authorship positions, and reported temporal improvement toward gender equity. When the authors compared the gender equity profile of publications according to pediatric discipline, there was a nearly universal pattern of female underrepresentation as senior authors. However, there was a temporal trend toward increasing representation as senior authors, which augurs gender equity in publication in the “near future.”

It is difficult to measure progress toward gender equity because there is no universal definition. Representation is an appealing proxy because it is quantifiable but it may not accurately reflect inclusion. Engagement in research and publication is an important dimension of scientific inclusion and the article by Boehm et al. suggests that publication could be considered an objective and reproducible measure of inclusion. Whereas authorship used to be assigned in an honorific fashion, organizations such as the International Committee of Medical Journal Editors have clearly defined the expectations and authors are expected to follow the recommendations. In Boehm et al.’s international study of 10 years of publications in pediatrics, male authors were more productive than female authors and nearly two-thirds of female authors published only one manuscript. The authors propose that the “leaking pipeline” contributes to the lack of productivity and paucity of women at senior rank. They suggest that gender-based differences in career aspirations and a lack of female role models also contribute to gender-based differences in publication. Promising Practices is the report from a recent consensus study undertaken by the National Academies of Sciences, Engineering and Medicine that highlighted the importance of institutional and structural barriers that affect many women in STEM fields, in addition to personal circumstances.^1^ The challenge of enhancing gender diversity along the scholarly continuum will require active engagement from individuals, leaders, institutions, and organizations. For example, institutions and organizations could establish or modify policies and programs in order to mitigate adverse impacts on career development. Recently, the University of California, San Francisco modified its policies related to family formation and expanded faculty members’ paid childbearing and childrearing leave to 12 weeks. After the initial phase of family formation, however, it may still be difficult for research-focused faculty members to balance the work-non-work continuum amidst the ever-changing needs and pressures associated with raising a family. Even if women experience more difficulty as junior and mid-career faculty members as compared to men, it may be dangerously inaccurate to assume that they are less committed to or invested in their careers. Organizations such as the Association of American Medical Colleges and Drexel University’s Executive Leadership in Academic Medicine have well-established professional development programs for female and underrepresented faculty members. Finally, while women are more likely to have part-time employment than men, the pandemic has catapulted many institutions and organizations into considering alternative models of employment, which may ultimately have positive or negative impacts on gender disparities.

For journal editors and editorial board members, several study findings are relevant. First, over the 10-year study period, there was no relationship between the proportion of female authors and the impact factor. At a time when we seek to understand the impact of diversity on organizational and institutional metrics of success, the results suggest that a temporal shift toward more female authors is not a major driver of impact factor. Since publication is the final step in a long scholarly pathway, future analyses could also consider the role of the journal selection, manuscript submission, and review processes, which include journal editors and manuscript reviewers. Williams et al. analyzed the role of gender in publication in *The Journal of Pediatrics*.^2^ Based on an analysis of all original research articles submitted to the journal over 2 consecutive years (2015–2016), there was no difference in reviewers’ recommendations or editors’ decisions based on the gender of the corresponding author. However, there were two gender-based differences in the publication process. First, women who were invited to review articles were less likely to accept the review invitation as compared to men; second, female editors had lower acceptance rates than male editors. Williams et al.’s results were similar to a previous study of articles submitted to *JAMA* in 1991.^3^ Taken together, the studies of these prestigious publications suggest that there is no systematic bias against studies submitted by female corresponding authors but that gender influences certain aspects of the review process. It is not clear if the editorial experience of these journals is representative and editors could analyze their own experience to assess whether there is any evidence of gender-based publication bias. For example, in the study by Boehm et al., *Pediatric Research* is approaching gender equity in authors (45.5%), the FAOR triplet profile was (+, =, –), and the Prestige Index was 0.0 (Table 2). Around the world, pediatric editorial boards may review their journal’s data profile, in addition to the standard measures of success and impact. For everyone who is “invited” to review manuscripts, I encourage you to actively engage in supporting the research process, which is founded on the principle of objective review by qualified experts.

To assess progress toward gender equity in scientific publishing and research, the use of name is an interesting measure because it is not fixed and may be an imprecise indicator of sex. Even with a sophisticated algorithm, one-quarter of authors initially selected for the study had “unisex” or non-identifiable names, which resulted in their exclusion from the study sample. Moreover, articles from China and South Korea had disproportionately high proportions of unisex names, which limits the interpretation of gender equity in those countries. In addition to the inherent limitations of algorithms that assign sex on the basis of name, the measure is binary, which excludes non-binary individuals and limits our interpretation of the findings. To address these issues, journals may consider asking authors to self-report sex and other demographic characteristics at the time of manuscript submission.

Boehm et al. documented, in their analysis of 10 years of publications, that women in pediatrics are highly engaged in research and publication. Asking and answering scientific questions is of fundamental importance to our profession, the “coin” of our realm, and the authors documented near-equity in gender representation among authors, with disproportionate representation among first authors. Given the dramatic temporal increase in women who have enrolled in medical school, it is encouraging that our community of research scholars has diversified, and we should be proud that pediatric researchers have achieved a greater level of gender diversity than many other medical disciplines. To monitor progress toward gender equity in scientific scholarship, publication metrics could be considered a biomarker and tracked by journal editors. The analysis of gender diversity at specific authorship positions suggests an impending “trickle over” toward senior authorship. A similar analytic approach could be employed to assess progress toward racial/ethnic equity in publication, as long as the data used to define gender, race/ethnicity or any other measure of diversity were self-reported, rather than assigned, to minimize inaccurate or misleading assignments. The path to a health professional or research career is long and the results by Boehm et al. suggest that publication metrics may be a useful “leading indicator” of equity and inclusion.
